# Expression Analysis of Macrodactyly Identifies Pleiotrophin Upregulation

**DOI:** 10.1371/journal.pone.0040423

**Published:** 2012-07-27

**Authors:** Frank H. Lau, Fang Xia, Adam Kaplan, Felecia Cerrato, Arin K. Greene, Amir Taghinia, Chad A. Cowan, Brian I. Labow

**Affiliations:** 1 Center for Regenerative Medicine and Cardiovascular Research Center, Massachusetts General Hospital, Boston, Massachusetts, United States of America; 2 Department of Plastic and Oral Surgery, Children’s Hospital Boston, Boston, Massachusetts, United States of America; Harvard Medical School, United States of America

## Abstract

Macrodactyly is a rare family of congenital disorders characterized by the diffuse enlargement of 1 or more digits. Multiple tissue types within the affected digits are involved, but skeletal patterning and gross morphological features are preserved. Not all tissues are equally involved and there is marked heterogeneity with respect to clinical phenotype. The molecular mechanisms responsible for these growth disturbances offer unique insight into normal limb growth and development, in general. To date, no genes or loci have been implicated in the development of macrodactyly. In this study, we performed the first transcriptional profiling of macrodactyly tissue. We found that pleiotrophin (PTN) was significantly overexpressed across all our macrodactyly samples. The mitogenic functions of PTN correlate closely with the clinical characteristics of macrodactyly. PTN thus represents a promising target for further investigation into the etiology of overgrowth phenotypes.

## Introduction

**Figure 1:**
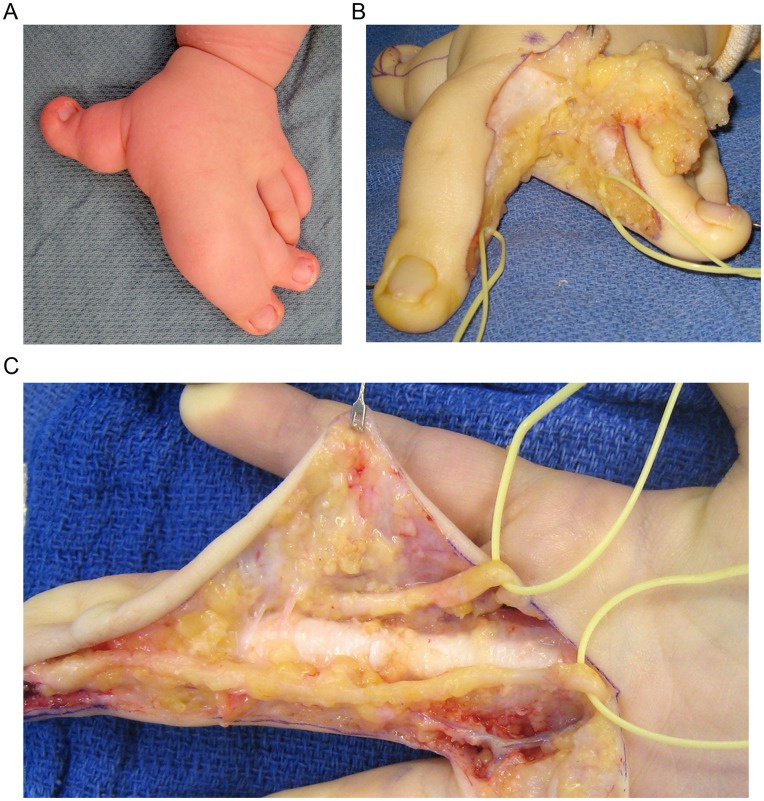
Clinical photos from patients undergoing surgical treatment of macrodactyly. (A) 15 month-old boy with macrodactyly involving the thumb, index and middle fingers. As is often seen, there is associated syndactyly between the index and middle fingers and deviation of all affected digits. (B) The same patient during separation and first stage debulking of the digits. The yellow loops are around the digital nerves, which are enlarged. A large volume of overgrown fat and soft-tissue is being removed. (C) 8 year-old girl with macrodactyly isolated to the middle finger. The excess fat and soft-tissue has been removed revealing enlarged digital nerves tagged with yellow loops.

Macrodactyly is characterized by the diffuse enlargement of 1 or more digits [1]–[3] (Fig. 1). It is a rare, congenital disease with no familial inheritance pattern, and usually presents as an isolated, non-syndromic condition. While all tissue elements are involved, including fat, skin, nerve, and bone, histopathologic analysis of macrodactyly tissue is usually remarkable for excess mature adipose tissue [3].

We currently possess no modern molecular insight into macrodactyly. No genes or loci have been implicated in the development of this disease. Neither transcriptional nor protein-level characterization of affected tissues is available. There are no cellular or animal models of macrodactyly.

In this study, we performed the first transcriptional profiling of macrodactyly tissue. We found that a developmental cytokine, pleiotrophin (PTN), was significantly overexpressed across all our macrodactyly samples. As a mitogen that has been implicated in nerve, bone, vascular, and tumor growth, the functions of PTN correlate closely with the clinical characteristics of macrodactyly. PTN thus represents a promising target for further investigation.

## Materials and Methods

This Project was reviewed and approved by the Children’s Hospital Boston Institutional Review Board, Assurance #07-11-0428 and by the Brigham & Women’s Hospital Institutional Review Board, Assurance # FWA00000484. Pediatric patients who underwent elective surgical debulkings of isolated, nonsyndromic macrodactyly were eligible for this study. Additionally, normal abdominal subcutaneous adipose tissue (SAT) samples were obtained from patients undergoing elective surgeries at either Massachusetts General Hospital or Children’s Hospital Boston. During the review of this Project, the IRB specifically considered (i) the risks and anticipated benefits, if any, to subjects; (ii) the selection of subjects; (iii) the procedures for securing and documenting informed consent; (iv) the safety of subjects; and (v) the privacy of subjects and confidentiality of the data. Written informed consent was obtained from guardians on the behalf of all minors/children participants.

Approximately 5 mg of adipose tissue were harvested from each patient and sectioned into smaller pieces. Samples were either snap frozen in liquid nitrogen and stored at −80C or fixed in either fresh 4% paraformaldehyde or 10% neutral buffered formalin (Sigma Aldrich) for 4 hours, transferred to phosphate buffered saline and stored in a 4 C refrigerator.

For each patient, RNA was extracted in biological triplicate via submersion in 1 ml of Trizol, mechanical lysis using an RNase free pestle (Kimble Chase Kontes), addition of 200 µl chloroform, and centrifugation (10,000 g, 15 minutes, 4 C). The supernatant was extracted and purified with RNeasy Mini Columns (Qiagen). 1.2 µg RNA was synthesized into complementary DNA (cDNA) with the Superscript III First Strand Synthesis Kit (Invitrogen).

All RNA samples were analyzed for quality using an Agilent 2100 Bioanalyzer. Hybridization to Affymetrix Human Genome U133 Plus 2.0 Arrays and subsequent array processing was done by Asuragen. All data is MIAME compliant and the raw data has been deposited in the Gene Expression Omnibus database (accession number GSE35820). Adult SAT datasets were found using the Expression Omnibus (GEO) [4] and ArrayExpress [5] databases by searching for the terms “adipose” and “fat”, and restricting the platform to Affymetrix Human Genome U133 Plus 2.0 Arrays.

Raw expression values were analyzed with the Partek Genomics Suite (Partek). Data were normalized using Robust Multiarray Averaging (RMA) with median scaling, quintile normalization, and background correction. The resulting datasets were preprocessed to remove probesets whose minimum fold change (maximum gene expression value divided by the minimum value) was <2, or whose difference between maximum and minimum values was less than 100. Prinicipal component analysis was performed, and linear models were used to identify statistically significant, differentially expressed probe sets. Hierarchical clustering was performed across all samples using the differentially expressed probesets.

GEA was performed using FuncAssociate 2.0 (http://llama.mshri.on.ca/funcassociate/), which uses a Fisher’s exact test to assess enrichment and a resampling approach to correct for multiple hypotheses. For each of the sample populations, a false discovery rate (FDR) of 0.01 was set as the threshold. The differentially expressed probesets were uploaded into FuncAssociate 2.0 as ordered lists. Analysis was performed using the hgnc_symbol namespace, with 1000 permutations for p-value estimation and a p-value cutoff of 0.05.

For qPCR, expression levels of PTN were normalized to the housekeeping gene hypoxanthine guanine phosphoribosyl transferase (HPRT) and measured via Quantifast SYBR Green PCR Kit (Qiagen). To minimize the potential impact of any genomic DNA contamination, primers were designed and verified to span multiple exons. Three technical replicates were performed for each sample. Error bars were computed by adding and subtracting 1 unit standard deviation of the delta Ct values from calibrated delta Ct values. P-values were calculated in Microsoft Excel 2007 using 2-tailed heteroscedastic Student’s t-tests.

For immunohistochemical staining, tissues samples fixed in freshly prepared 4% paraformaldehyde were dehydrated using an ethanol gradient, sectioned (5-micron thickness), and mounted on glass slides (Fisher). The sections were rehydrated and antigen retrieval was performed using citric acid buffer (pH 6.0). The sections were blocked using 5% donkey serum (Jackson Labs) with 0.01% Triton, and incubated with goat polyclonal anti-pleiotrophin antibody (1∶100 dilution, Abcam ab10849). Bound anti-PTN antibody was visualized using Alexa Fluor® 546 donkey anti-goat IgG (H+L) secondary antibody 1∶700 (Invitrogen A-11056). Images were captured using a Nikon Eclipse Ti-s.

DNA was isolated from our samples using a DNeasy Blood & Tissue Kit (Qiagen). The isolates were analyzed for quality using an Agilent 2100 Bioanalyzer. Sequencing of the promoter and coding regions of PTEN was performed by Polymorphic DNA Technologies.

## Results

Between June 2009 and April 2011, 4 pediatric patients (Patients 1 through 4) who underwent elective surgical debulkings of isolated, nonsyndromic macrodactyly participated in the study. Normal abdominal SAT from 2 adult patients (Patients 5 & 6) who underwent elective surgeries at Massachusetts General Hospital between June 2009 and March 2010 was obtained. Normal abdominal SAT was also obtained from 1 pediatric patient (Patient 7) who underwent an elective abdominal procedure at Children’s Hospital Boston.

RNA was isolated from the 4 macrodactyly adipose tissue samples and hybridized to Affymetrix U133A Plus 2.0 microarrays. We sought to compare our samples against publicly available data sets; since macrodactyly tissues are usually identified as “mature adipose tissue” on histopathologic examination, we chose subcutaneous adipose tissue (SAT) datasets as reference data sets. No suitable data was available in the Gene Expression Omnibus (GEO) [4] and ArrayExpress [5] databases for pediatric samples, thus we performed our analysis against 4 adult SAT datasets comprising 345 patients. Datasets GSE135063 [6], GSE171704 [7], GSE157735 [8] were pooled and referred to as the “GSM” pool. Dataset E-TABM-325 [8] was referred to as the “MolPAGE” pool.

**Figure 2:**
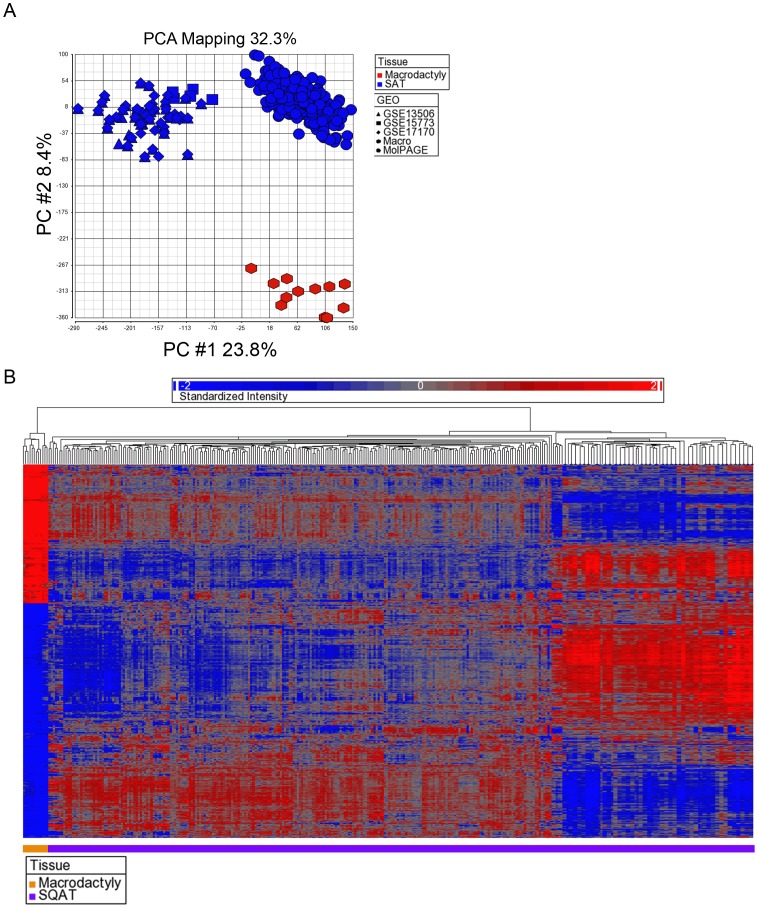
Results of principal component analysis (PCA) and hierarchical clustering of gene expression date from macrodactyly samples. (A) PCA of 4 macrodactyly samples (in triplicate) vs. 345 subcutaneous adipose tissue (SAT) samples. The top 2 vectors account 32.3% of intersample variation. Macrodactyly samples cluster distinctly from SAT. (B) Hierarchical clustering of macrodactyly and SAT samples across differentially expressed genes with fold-change >1.5, p-value <0.05, and false discovery rate <0.05. Macrodactyly samples cluster distinctly and distantly from all SAT samples.

To analyze these data, we performed principal component analysis (Fig. 2A). The top 2 vectors accounted for 23.8% and 8.5% of inter-sample variation. This analysis demonstrated clear separation between macrodactyly and SAT samples. We next developed transcriptional profiles of macrodactyly and SAT. We restricted our analysis to probe sets with false-discovery rates (FDR) <0.01, fold-changes ≥1.5, and p-values <0.05. With these stringent restrictions, we identified 3093 overexpressed and 4202 underexpressed genes. The large number of differentially expressed genes highlights the stark differences between macrodactyly and normal adipose tissue. The 10 features with the highest fold-changes are shown in Table 1. When hierarchical clustering was performed using these 7295 differentially expressed genes, the macrodactyly samples clustered distinctly and distantly from all SAT samples (Fig. 2B).

**Table 1:** Top 10 greatest fold-change probesets in macrodactyly and subcutaneous adipose tissue.

Overexpressed in Macrodactyly				
Probeset ID	Gene	Description	p-value	Fold-Change (vs. SAT)
209465_x_at	PTN	pleiotrophin	0	34.44
223475_at	CRISPLD1	cysteine-rich secretory protein LCCL domain containing 1	1.85E-42	21.42
211737_x_at	PTN	pleiotrophin	0	19.86
220504_at	KERA	keratocan	0	18.51
203913_s_at	HPGD	hydroxyprostaglandin dehydrogenase 15-(NAD)	1.40E-45	15.71
216834_at	RGS1	regulator of G-protein signaling 1	2.72E-25	14.19
205430_at	BMP5	bone morphogenetic protein 5	0	13.97
209466_x_at	PTN	pleiotrophin	0	12.97
209189_at	FOS	FBJ murine osteosarcoma viral oncogene homolog	3.03E-15	12.82
203700_s_at	DIO2	deiodinase, iodothyronine, type II	0	12.35
**Overexpressed in SAT**				
**Probeset ID**	**Gene**	**Description**	**p-value**	**Fold-Change (vs. Macrodactyly)**
214456_x_at	SAA1///SAA2	serum amyloid A1///serum amyloid A2	4.20E-45	91.68
208607_s_at	SAA1///SAA2	serum amyloid A1///serum amyloid A2	4.84E-26	72.59
204424_s_at	LMO3	LIM domain only 3 (rhombotin-like 2)	0	17.56
214146_s_at	PPBP	pro-platelet basic protein (chemokine (C-X-C motif) ligand 7)	1.72E-21	16.27
228434_at	BTNL9	butyrophilin-like 9	0	14.31
211699_x_at	HBA1///HBA2	hemoglobin, alpha 1///hemoglobin, alpha 2	0	14.28
209458_x_at	HBA1///HBA2	hemoglobin, alpha 1///hemoglobin, alpha 2	0	13.49
204018_x_at	HBA1///HBA2	hemoglobin, alpha 1///hemoglobin, alpha 2	0	12.66
204105_s_at	NRCAM	neuronal cell adhesion molecule	2.39E-27	12.49
229778_at	C12orf39	chromosome 12 open reading frame 39	1.78E-09	12.35

To better characterize the differentially expressed transcripts, we performed unbiased gene enrichment analysis (GEA) using FuncAssociate 2.0. [9] The 2 tissue types were each enriched for different gene ontology categories (Table 2). In macrodactyly, there was an enrichment of growth factor response (Table S1), extracellular matrix (Table S2), and patterning (Table S3) genes. In contrast, SAT was enriched for classic adipose tissue gene categories such as regulation of fatty acid oxidation and response to insulin (Table 2).

**Table 2:** Enriched gene ontology categories in macrodactyly and subcutaneous adipose tissue.

# of genes	P-adjusted	GO ID	GO Category
***Upregulated in Macrodactyly***			
6	0.001	GO:0071363	cellular response to growth factor stimulus
6	0.039	GO:0070848	response to growth factor stimulus
10	0.048	GO:0030199	collagen fibril organization
18	0.011	GO:0001501	skeletal system development
19	0	GO:0005578	proteinaceous extracellular matrix
22	0	GO:0031012	extracellular matrix
30	0	GO:0005539	glycosaminoglycan binding
34	0	GO:0001871	pattern binding
34	0	GO:0030247	polysaccharide binding
41	0	GO:0005615	extracellular space
41	0	GO:0009611	response to wounding
42	0.01	GO:0008284	positive regulation of cell proliferation
47	0	GO:0048731	system development
48	0.008	GO:2000026	regulation of multicellular organismal development
49	0.023	GO:0005102	receptor binding
51	0	GO:0044421	extracellular region part
52	0.023	GO:0009653	anatomical structure morphogenesis
59	0	GO:0007166	cell surface receptor linked signaling pathway
77	0	GO:0005576	extracellular region
***Upregulated in Subcutaneous Adipose Tissue***			
3	0.002	GO:0015671	oxygen transport
3	0.008	GO:0005833	hemoglobin complex
3	0.012	GO:0015669	gas transport
3	0.012	GO:0005344	oxygen transporter activity
5	0	GO:0004556	alpha-amylase activity
5	0	GO:0016160	amylase activity
6	0.044	GO:0003823	antigen binding
12	0.013	GO:0003995	acyl-CoA dehydrogenase activity
17	0	GO:0046320	regulation of fatty acid oxidation
24	0.023	GO:0032869	cellular response to insulin stimulus
36	0.004	GO:0032868	response to insulin stimulus
43	0	GO:0001525	angiogenesis
55	0.013	GO:0051056	regulation of small GTPase mediated signal transduction
57	0.04	GO:0035467	negative regulation of signaling pathway
60	0.049	GO:0006732	coenzyme metabolic process
71	0.01	GO:0051270	regulation of cellular component movement
87	0.004	GO:0009894	regulation of catabolic process
96	0	GO:0005083	small GTPase regulator activity
123	0.004	GO:0030695	GTPase regulator activity
124	0.007	GO:0060589	nucleoside-triphosphatase regulator activity
197	0.015	GO:0046907	intracellular transport
204	0.012	GO:0019899	enzyme binding
222	0.04	GO:0035466	regulation of signaling pathway
234	0	GO:0044248	cellular catabolic process

**Figure 3:**
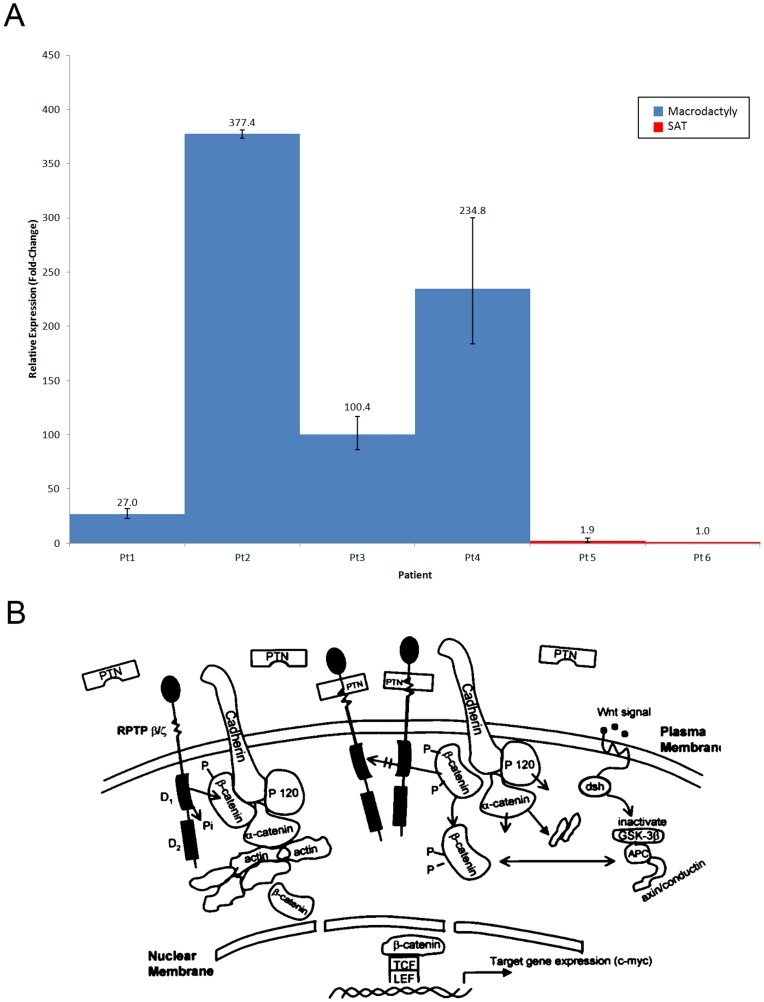
Confirmation of pleiotrophin (PTN) expression in macrodactyly and the known PTN signaling cascade. (A) Relative expression of PTN in macrodactyly vs. adult subcutaneous adipose tissue as determined by quantitative real time polymerase chain reaction. In macrodactyly, PTN averaged 127.6-fold overexpression (p = 0.049). (B) The PTN signaling cascade and crosstalk with Wnt signalling (from Deuel et al.)^14^.

PTN was the most highly overexpressed gene in macrodactyly (34.4-fold overexpression, p-value = 0.00). The developmental cytokine pleiotrophin was present in both the extracellular space and pattern binding GO annotation categories (Tables S2 & S3). To confirm these findings, we performed quantitative real time polymerase chain reaction (qPCR) and found that in macrodactyly, PTN averaged 127.6-fold overexpression (p = 0.049, Fig. 3A).

**Figure 4:**
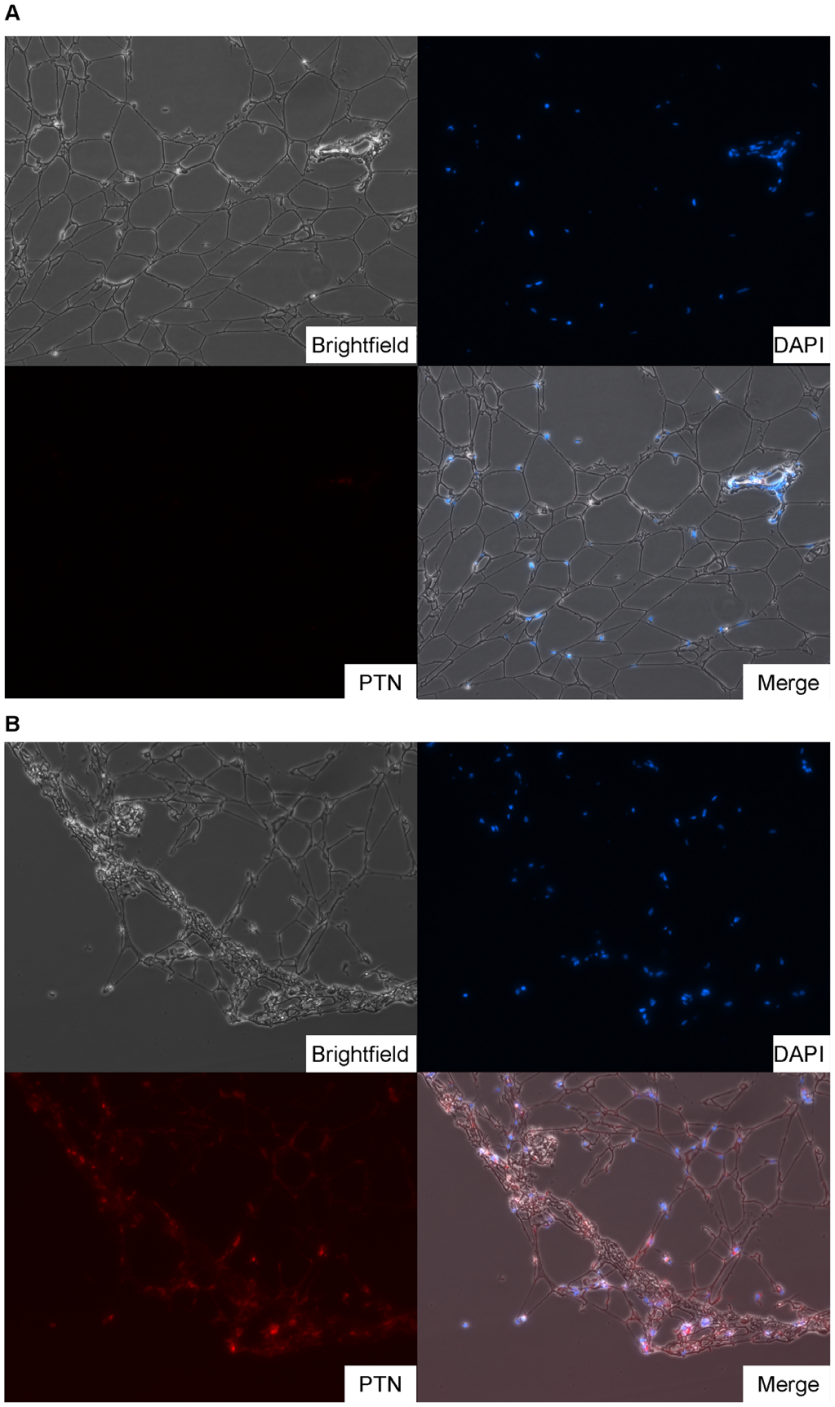
Pleiotrophin (PTN) immunostaining. DAPI  =  nuclear stain, PTN  =  antibody staining for pleiotrophin, Merge  =  composite image merging Brightfield, DAPI, and PTN channels. A) No PTN is seen in pediatric subcutaneous adipose tissue from Patient 7. B) Large PTN aggregates are seen in macrodactyly patient 4.

*In vivo*, PTN is secreted into the extracellular space where it binds to heparin. To demonstrate that transcriptional upregulation of PTN resulted into protein overproduction, we analyzed sections from adipose tissue from a patient with macrodactyly (Patient 4) and pediatric SAT (Patient 6) tissue for PTN immunoreactivity. We found no PTN staining in pediatric SAT (Fig. 4A). In contrast, macrodactyly sections were marked by widespread punctate expression of PTN (Fig. 4B). This assay did not allow the localization of PTN expression to a specific cell type. We attempted to quantify PTN overexpression by Western blot [10], [11] but the small volume of our samples made this technically infeasible. Li et al. reported that deletion of phosphatase and tensin homologue (PTEN) was associated with PTN upregulation [12]. We thus isolated DNA from our macrodactyly samples and sequenced the PTEN promoter and coding regions. No mutations were identified (data not shown).

## Discussion

Macrodactyly remains a difficult clinical problem, one with an unknown etiology. Modern treatment strategies are strictly surgical and involve either growth-limiting or digit reducing procedures, or, in severe cases, finger amputation [2], [3], [13]. Prior to this study, no molecular information was available regarding this disease.

Given the absence of molecular data, we elected to first generate transcriptional profiles of macrodactyly tissue. Analysis of this data identified 7295 differentially expressed genes in macrodactyly compared to adult SAT. The candidate genes overexpressed in macrodactyly include well-characterized mitogens such as bone-morphogenetic proteins 5 & 7, transforming growth factor beta 3, and Wnt signaling pathway members (WNT2, WNT5A) (Tables S1 & S2). However, the mitogen with the highest fold-change overexpression was pleiotrophin (Table 1).

To characterize our samples at the biological pathway level, we performed gene enrichment analysis. This again underscored the differences between SAT and macrodactyly: SAT was enriched for classic adipose tissue GO categories such as “regulation of catabolic processes” and “response to insulin stimulus” (Table 2). In contrast, macrodactyly tissues demonstrated enrichment of the gene ontology (GO) categories such as “extracellular space” and “pattern binding” (Table 2).

Interestingly, PTN was present in both of these GO categories (Tables S2 & S3). This fact, combined with its rank as the gene with the greatest fold-change overexpression on microarray analysis, led us to perform confirmatory experiments on this candidate gene. At the transcriptional level, qPCR confirmed PTN overexpression in macrodactyly compared to adult abdominal SAT, a depot known for its remarkable proliferative capacity (Fig 3A). While PTN was overexpressed in all macrodactyly samples, the degree of overexpression varied greatly between patients, with overexpression levels being lowest in patient 1. There were insufficient samples to correlate PTN overexpression levels with clinical phenotype, but it is possible that lower overexpression corresponds with milder disease. At the protein level, immunohistochemical staining revealed punctate aggregates of PTN in macrodactyly (Fig. 4B). Control pediatric finger adipose tissue from a patient with polydactyly demonstrated no PTN staining (Fig. 4A).

Pleiotrophin is a promising candidate gene for the pathogenesis of macrodactyly because it promotes growth of nearly all the tissues affected by macrodactyly, including nerve, skin, bone, and cartilage. An 18-kDa protein, PTN was the first developmentally regulated cytokine to be discovered [14]. Its amino acid sequence is the most highly conserved between human, bovine, rat, mouse, and chicken cytokines [15]. It possesses 50% sequence homology with midkine [11], [15].

Pleiotrophin has been variously referred to as heparin-binding growth-associated molecule (HB-GAM), heparin-binding neurite outgrowth-promoting factor 1 (HBNF1), and osteoblast-stimulating factor 1 (OSF-1). This variety of names reflects its diverse mitogenic functions. In neural cells, PTN is necessary for proper neurite outgrowth and maturation in the central nervous system [14], [16], [17]. In the peripheral nervous system it promotes nerve regeneration following injury [11]. PTN is highly expressed *in vivo* in bone and cartilage, and is upregulated in response to mechanical loading [18], [19], [20]. As an angiogenic factor, PTN supports endothelial cell proliferation [21]. PTN has also been implicated in a number of tumors including glioblastoma [22], [23] and breast cancer [24], [25], and its overexpression has been reported to cause malignant transformation in several cell lines [15], [26]. While one study suggested that PTN inhibits adipogenesis, the findings were indirect and only used an *in vitro* model [27].

The overlap between the physiologic functions of PTN and the clinical phenotype of macrodactyly is striking. In neural cells, PTN directs nerve growth and regeneration; clinicians have long noted that macrodactyly nerves are unusually large [3], [28], [29]. PTN has been demonstrated to be osteogenic and chondrogenic; one of the hallmarks of macrodactyly is persistent bone and joint overgrowth. As a protooncogene, PTN drives fibroblast, endothelial cell, and epithelial cell growth; all of these soft tissue elements are overgrown in macrodactyly. In many patients, macrodactylous overgrowth is proportionally patterned among all tissues of the affected region. The means by which this specific, patterned overgrowth occurs is not known.

One mechanism whereby PTN overexpression might result in macrodactyly was recently suggested by the discovery of an activating AKT1 mutation in Proteus syndrome [30]. This mutation leads to constitutive phosphorylation of residues Ser473 and Thr308 in AKT1. PTN, on the other hand, has been shown to rapidly phosphorylate Ser473 of AKT1 in a dose-dependent manner [31]. This link is particularly intriguing because partial gigantism of the hands and/or feet is a hallmark of Proteus syndrome [32]. Unfortunately, phosphorylation of this specific residue is also consistent with rapid proliferation and thus, the use of this biomarker would not be conclusive as to mechanism of overgrowth. For this reason, testing for phosphorylation of residue Ser473 in AKT1 was not performed.

Little is currently known about the regulation of PTN. While it has been reported that PTEN deletion is associated with PTN upregulation, this is indirect and the direct regulation of PTN stemming from PTEN deletion is unknown [12]. Our microarray data did not demonstrate reduction of PTEN levels in macrodactyly (data not shown) and sequencing of the PTEN locus in macrodactyly samples yielded no mutations.

This study was limited by the unavailability of normal pediatric finger adipose tissue. Only under rare circumstances would it be ethical to remove a significant amount of tissue from a child’s hand for research purposes. Some pediatric hand conditions, such as polydactyly, are managed by finger amputation and therefore are potential sources of pediatric finger adipose tissue. However, these conditions can be caused by germline genetic mutations and therefore are not strictly normal [33]. Because of these limitations, we believe future research will require *in vitro* and animal models of macrodactyly. The identification of PTN as the first macrodactyly candidate gene points the way towards the development of critical research tools.

## Supporting Information

Table S1 **Genes present in the “Response to Growth Factor Stimulus (GO:0070848)” gene ontology category.**
(DOCX)

Table S2 **Genes present in the “Extracellular Space (GO:0005615)” gene ontology category.**
(DOCX)

Table S3 **Genes present in the “Pattern Binding (GO:0001871)” gene ontology category.**
(DOCX)
