# Supplementary material for: Expression Analysis of Macrodactyly Identifies Pleiotrophin Upregulation
Source: PLoS One. 2012 Jul 27;7(7):e40423. doi: 10.1371/journal.pone.0040423 (PMC3407187; doi:10.1371/journal.pone.0040423)
Supplement: Table S1 — Genes present in the “Response to Growth Factor Stimulus (GO:0070848)” gene ontology category. (DOCX) [file pone.0040423.s001.docx]

Table S1

| **Gene** | **p-value** | **Fold change** |
| --- | --- | --- |
| COL1A1 | 1.04E-12 | 5.34 |
| TGFB3 | 0 | 4.37 |
| WNT2 | 0 | 4.21 |
| WNT5A | 1.40E-45 | 3.95 |
| FEZ1 | 1.18E-27 | 3.47 |
| CTNNB1 | 4.77E-09 | 3.28 |
