# Supplementary material for: Expression Analysis of Macrodactyly Identifies Pleiotrophin Upregulation
Source: PLoS One. 2012 Jul 27;7(7):e40423. doi: 10.1371/journal.pone.0040423 (PMC3407187; doi:10.1371/journal.pone.0040423)
Supplement: Table S2 — Genes present in the “Extracellular Space (GO:0005615)” gene ontology category. (DOCX) [file pone.0040423.s002.docx]

| **Gene** | **p-value** | **Fold change** |
| --- | --- | --- |
| PTN | 0 | 34.44 |
| BMP5 | 0 | 13.97 |
| LRRC17 | 1.30E-16 | 10.94 |
| OGN | 9.76E-19 | 10.17 |
| DKK3 | 1.90E-24 | 9.72 |
| SFRP4 | 2.05E-11 | 7.04 |
| ANGPTL1 | 0 | 6.60 |
| VCAN | 3.62E-14 | 5.98 |
| COL14A1 | 2.05E-14 | 5.67 |
| PTHLH | 0 | 5.44 |
| IL8 | 2.83E-26 | 5.36 |
| COL1A1 | 1.04E-12 | 5.34 |
| DKK2 | 2.24E-19 | 5.07 |
| SCRG1 | 2.07E-27 | 4.74 |
| IL17D | 6.03E-44 | 4.47 |
| COMP | 2.96E-26 | 4.41 |
| TGFB3 | 0 | 4.37 |
| SOSTDC1 | 2.55E-34 | 4.22 |
| WNT5A | 1.40E-45 | 3.95 |
| IL33 | 1.94E-10 | 3.71 |
| FGF7 | 3.45E-29 | 3.65 |
| CTSG | 3.48E-17 | 3.54 |
| SULF1 | 2.06E-07 | 3.50 |
| EGFR | 1.98E-19 | 3.44 |
| C1QTNF2 | 2.51E-13 | 3.42 |
| SERPINE2 | 1.02E-16 | 3.35 |
| CTSK | 5.72E-16 | 3.34 |
| COL1A2 | 2.08E-13 | 3.24 |
| CCL3 | 1.27E-08 | 3.01 |
| CCL3L1 | 1.27E-08 | 3.01 |
| CCL3L3 | 1.27E-08 | 3.01 |
| TFPI | 6.52E-14 | 2.99 |
| DCN | 1.46E-15 | 2.99 |
| CCL13 | 2.68E-14 | 2.93 |
| CFH | 1.90E-21 | 2.89 |
| TNFSF13B | 2.90E-24 | 2.80 |
| CTGF | 1.86E-06 | 2.74 |
| NUCB2 | 3.80E-40 | 2.72 |
| ABI3BP | 3.41E-08 | 2.63 |
| LY96 | 1.27E-18 | 2.63 |
| FGF18 | 6.72E-16 | 2.61 |
| MPO | 2.80E-45 | 2.52 |
| DLK1 | 0 | 2.51 |
| SERPINE1 | 4.47E-24 | 2.49 |
| MYOC | 1.06E-09 | 2.42 |
| COL3A1 | 1.18E-13 | 2.42 |
| KIT | 2.02E-11 | 2.33 |
| FN1 | 8.83E-08 | 2.32 |
| FRZB | 7.94E-05 | 2.32 |
| LOXL4 | 7.54E-42 | 2.26 |
| SPP1 | 4.73E-04 | 2.26 |
| PCSK6 | 6.78E-13 | 2.26 |
| LUM | 3.64E-25 | 2.24 |
| HGF | 1.53E-19 | 2.23 |
| CCL2 | 2.51E-06 | 2.23 |
| FGF1 | 1.22E-04 | 2.21 |
| TNFSF4 | 4.69E-19 | 2.17 |
| SFRP2 | 1.96E-04 | 2.15 |
| CCL18 | 4.84E-04 | 2.13 |
| IL1RL1 | 9.81E-45 | 2.13 |
| CFHR1 | 1.78E-12 | 2.12 |
| STC1 | 4.67E-07 | 2.05 |
| KITLG | 2.18E-08 | 2.04 |
| SFN | 9.43E-05 | 2.02 |
| BCHE | 1.97E-04 | 1.98 |
| TWSG1 | 1.67E-24 | 1.93 |
| IFNA17 | 5.02E-37 | 1.93 |
| TGFBI | 1.45E-06 | 1.92 |
| IL7 | 3.76E-11 | 1.89 |
| BMP7 | 1.38E-16 | 1.89 |
| CKLF | 2.24E-20 | 1.87 |
| CXCL2 | 2.12E-03 | 1.87 |
| ASIP | 1.68E-44 | 1.87 |
| MIF | 4.82E-20 | 1.86 |
| PXDNL | 3.01E-23 | 1.86 |
| HSPD1 | 6.31E-22 | 1.83 |
| MGP | 1.12E-10 | 1.82 |
| CLEC3B | 2.49E-13 | 1.79 |
| CRLF1 | 8.54E-11 | 1.77 |
| IGFBP6 | 2.24E-04 | 1.76 |
| GDF11 | 2.00E-29 | 1.75 |
| FBLN1 | 1.10E-26 | 1.75 |
| ADAM9 | 2.16E-03 | 1.75 |
| CCL4 | 5.41E-07 | 1.74 |
| SEMA3C | 4.61E-03 | 1.73 |
| FAM3B | 7.35E-09 | 1.72 |
| HMGB1 | 4.76E-44 | 1.72 |
| IL6ST | 5.70E-04 | 1.71 |
| TNF | 2.95E-25 | 1.71 |
| IL19 | 2.91E-23 | 1.69 |
| TAC3 | 9.64E-29 | 1.68 |
| BMP15 | 1.85E-42 | 1.67 |
| DEFA6 | 5.87E-27 | 1.66 |
| TNFSF9 | 4.97E-16 | 1.66 |
| CTSZ | 1.78E-03 | 1.66 |
| IL1B | 4.69E-18 | 1.65 |
| SCG2 | 3.30E-11 | 1.65 |
| ERBB3 | 1.54E-12 | 1.64 |
| FGF10 | 1.78E-03 | 1.64 |
| PRSS33 | 3.99E-29 | 1.64 |
| NDP | 1.28E-17 | 1.62 |
| AFP | 1.19E-21 | 1.62 |
| IL18 | 4.41E-04 | 1.62 |
| IFNA7 | 1.06E-27 | 1.61 |
| GSN | 4.33E-10 | 1.61 |
| GH1 | 8.03E-32 | 1.61 |
| SRGN | 3.27E-12 | 1.61 |
| SPN | 9.17E-27 | 1.60 |
| PSPN | 3.38E-15 | 1.60 |
| PMCH | 4.91E-16 | 1.59 |
| IGFBP7 | 4.31E-05 | 1.59 |
| MASP1 | 2.27E-33 | 1.58 |
| AKR1B1 | 2.10E-18 | 1.57 |
| VCAM1 | 3.79E-04 | 1.56 |
| FBN1 | 5.10E-04 | 1.55 |
| SERPINA1 | 9.79E-20 | 1.55 |
| IL28A | 1.32E-20 | 1.55 |
| PCOLCE | 5.59E-04 | 1.55 |
| IL13RA2 | 2.08E-14 | 1.55 |
| FGFBP1 | 5.70E-20 | 1.54 |
| BMP8B | 2.43E-04 | 1.54 |
| CCL27 | 5.60E-06 | 1.53 |
| UTS2 | 1.05E-07 | 1.53 |
| HPX | 3.03E-23 | 1.52 |
| SLIT1 | 3.02E-17 | 1.52 |
| IL29 | 9.51E-25 | 1.52 |
| IFNA14 | 1.26E-15 | 1.52 |
| TFF1 | 1.97E-16 | 1.51 |
| CNTF | 1.02E-23 | 1.51 |
| MEP1B | 2.01E-21 | 1.51 |
| HYAL1 | 8.79E-08 | 1.51 |
| CD109 | 5.60E-11 | 1.51 |
