# Supplementary material for: Expression Analysis of Macrodactyly Identifies Pleiotrophin Upregulation
Source: PLoS One. 2012 Jul 27;7(7):e40423. doi: 10.1371/journal.pone.0040423 (PMC3407187; doi:10.1371/journal.pone.0040423)
Supplement: Table S3 — Genes present in the “Pattern Binding (GO:0001871)” gene ontology category. (DOCX) [file pone.0040423.s003.docx]

| **Gene** | **p-value** | **Fold change** |
| --- | --- | --- |
| PTN | 0 | 34.44 |
| TNFAIP6 | 1.98E-28 | 8.46 |
| LYVE1 | 3.54E-12 | 6.55 |
| VCAN | 3.62E-14 | 5.98 |
| FGF7 | 3.45E-29 | 3.65 |
| COMP | 2.96E-26 | 4.41 |
| BGN | 4.34E-23 | 3.88 |
| CTSG | 3.48E-17 | 3.54 |
| SERPINE2 | 1.02E-16 | 3.35 |
| PRG4 | 5.1E-08 | 3.13 |
| DCN | 1.46E-15 | 2.99 |
| PLA2G5 | 1.19E-13 | 2.99 |
| CTGF | 1.86E-06 | 2.74 |
| APP | 1.61E-11 | 2.66 |
| MPO | 2.8E-45 | 2.52 |
| CCDC80 | 5.75E-13 | 2.51 |
| GPNMB | 8.5E-10 | 2.50 |
| CLEC7A | 7.35E-06 | 2.42 |
| FN1 | 8.83E-08 | 2.32 |
| PCSK6 | 6.78E-13 | 2.26 |
| CCL2 | 2.51E-06 | 2.23 |
| PTPRC | 3.48E-06 | 2.21 |
| FGF1 | 0.000122 | 2.21 |
| CD44 | 4.67E-05 | 2.18 |
| ITGAM | 5.19E-10 | 2.09 |
| MAMDC2 | 2.26E-07 | 2.08 |
| C8orf84 | 1.52E-08 | 2.04 |
| FGF14 | 2.3E-34 | 1.99 |
| HMMR | 1.98E-41 | 1.97 |
| NLRP3 | 1.16E-10 | 1.95 |
| LAYN | 5.49E-13 | 1.92 |
| COL5A1 | 1.93E-08 | 1.91 |
| PTX3 | 0.00086 | 1.90 |
| BMP7 | 1.38E-16 | 1.89 |
| C4orf31 | 1.18E-06 | 1.86 |
| PGLYRP4 | 4.8E-22 | 1.74 |
| TINAG | 4.56E-29 | 1.69 |
| RPL22 | 3.54E-28 | 1.67 |
| STAB2 | 4.34E-18 | 1.66 |
| FGF10 | 0.001782 | 1.64 |
| BCAN | 9.11E-32 | 1.64 |
| EPYC | 2.44E-23 | 1.60 |
| ADAMTS1 | 0.001148 | 1.59 |
| ZNF146 | 0.001154 | 1.57 |
| COL13A1 | 0.000385 | 1.56 |
| FGFBP1 | 5.7E-20 | 1.54 |
| FGF4 | 4.86E-21 | 1.52 |
